# Treatment patterns and clinical outcomes in chronic myeloid leukemia treated with second-line nilotinib—association of molecular milestones and prognostic scores with deep molecular response: a real-world retrospective cohort study

**DOI:** 10.1590/1516-3180.2026.3647.06052026

**Published:** 2026-07-20

**Authors:** Irena Ćojbašić, Ivan Tijanić, Žarko Ćojbašić

**Affiliations:** I Faculty of Medicine, University of Niš, Niš, Serbia.; II Faculty of Medicine, University of Niš, Niš, Serbia.; III Faculty of Mechanical Engineering, University of Niš, Niš, Serbia.

**Keywords:** Leukemia, myelogenous, chronic, BCR-ABL, positive, Nilotinib, Prognosis, Risk assessment, Treatment outcome, Deep molecular remission, European LeukemiaNet milestones, Treatment-free remission

## Abstract

**BACKGROUND:**

Real-world data on the treatment patterns and clinical outcomes of second-line nilotinib use in chronic myeloid leukemia (CML) remain limited, particularly regarding predictors of deep molecular response (DMR).

**OBJECTIVES:**

To characterize treatment patterns and long-term clinical outcomes in patients with CML receiving second-line nilotinib and to evaluate whether early molecular milestones combined with baseline prognostic scores predict DMR.

**DESIGN AND SETTING:**

Retrospective, single-center real-world study conducted at a tertiary academic center between 2011 and 2025.

**METHODS:**

The data of 45 patients with CML receiving second-line nilotinib because of imatinib resistance or intolerance were analyzed. Treatment patterns, including duration and reasons for switching therapy, were assessed. Baseline prognostic scores (Sokal, European Treatment and Outcome Study [EUTOS], EUTOS Long-Term Survival [ELTS], Hammersmith) were calculated, and molecular responses were assessed according to the European LeukemiaNet (ELN) criteria. Predictors of DMR were examined using univariate and multivariate logistic regression, and survival outcomes were estimated using Kaplan–Meier analysis.

**RESULTS:**

The median duration of nilotinib administration was 67.3 months, with 60% of patients achieving a best response of DMR and 46.7% maintaining a sustained DMR. Univariate analysis showed that a favorable Hammersmith score (p = 0.001), low ELTS risk (p = 0.028), and attainment of ELN-defined milestones at 6 (p = 0.020) and 12 months (p = 0.006) were significantly associated with DMR. Multivariate analysis demonstrated that the 12-month ELN milestone (p = 0.008) and favorable Hammersmith score (p = 0.023) were independently associated with a DMR. Patients achieving a DMR showed a nonsignificant trend toward improved overall survival.

**CONCLUSION:**

In this real-world cohort, a DMR and favorable survival outcomes were achieved when nilotinib was administered as second-line treatment. Integrating baseline prognostic scores with molecular milestones enables individualized monitoring and supports a sustained DMR as a marker of durable disease control.

## INTRODUCTION

Chronic myeloid leukemia (CML) is a clonal myeloproliferative neoplasm driven by the constitutively active *BCR::ABL1* (breakpoint cluster region::Abelson 1) tyrosine kinase generated by the Philadelphia chromosome, t(9;22)(q34;q11).^1^ Quantitative monitoring of *BCR::ABL1* transcripts is central to managing CML, enabling early identification of patients at risk of suboptimal response and informing therapeutic decision-making.^2^ High rates of cytogenetic and molecular responses are achieved with the use of the first-generation tyrosine kinase inhibitor (TKI) imatinib and second-generation TKIs, including nilotinib, dasatinib, and bosutinib.^3,4,5^

Despite these advances, a subset of patients discontinues first-line therapy due to resistance or intolerance and requires second-line treatment.^3,4,5^ Treatment failure is frequently associated with *BCR::ABL1* kinase domain mutations and other resistance mechanisms, whereas intolerance reflects clinically significant treatment-related toxicities necessitating that therapy be modified or switched.^5^ Both clinical trials and real-world studies have demonstrated that second-line treatment with nilotinib induces durable molecular responses and favorable survival outcomes.^6,7,8^

Although *BCR::ABL1* transcript kinetics are well established for response monitoring, predictors of deep molecular response (DMR) in the second-line setting remain incompletely defined. DMR reflects profound and sustained suppression of *BCR::ABL1* transcripts; it is associated with improved long-term outcomes, such as progression-free survival (PFS) and the potential for treatment-free remission, confirming its clinical relevance as a therapeutic endpoint.^6,7,8^ European LeukemiaNet (ELN)-defined molecular milestones at 6 and 12 months provide dynamic response assessment, and baseline prognostic scoring systems—including the Sokal, European Treatment and Outcome Study (EUTOS), EUTOS Long-Term Survival (ELTS), and Hammersmith systems—offer complementary information on disease risk.^9,10,11,12^ However, most studies have evaluated these parameters separately, and data integrating static baseline risk with molecular dynamics during treatment to predict the DMR in patients receiving second-line nilotinib remain limited.

## OBJECTIVE

In this study, we aimed to evaluate whether integrating 6- and 12-month ELN-defined molecular milestones with baseline prognostic scores improved prediction of the DMR in a real-world cohort of patients with chronic-phase CML who were undergoing second-line nilotinib treatment, thereby supporting risk-adapted monitoring and individualized clinical decision-making in routine practice.

## METHODS

### Study design and population

In this retrospective, real-world study, data from a database of patients with chronic-phase CML were analyzed to evaluate treatment patterns and molecular outcomes at a single center between 2011 and 2025. In total, 45 patients received nilotinib as second-line treatment because they were resistant or intolerant to imatinib. The small sample size reflects the rarity of second-line use of nilotinib in routine practice, highlighting the real-world nature of the cohort. Nilotinib was administered at the standard approved dose of 800 mg/day. Primary resistance was defined as failure to achieve an adequate cytogenetic or molecular response within the expected timeframe. Secondary resistance was defined as the loss of a previously achieved response.

### Baseline prognostic assessment

Baseline prognostic scores were calculated at diagnosis to stratify patients according to their expected clinical outcomes and likelihood of responding to TKI therapy. The Sokal score was used to predict overall survival (OS), with patients stratified into low, intermediate, and high risk at diagnosis, categories. Using the EUTOS score to estimate the probability of a cytogenetic response, patients were classified as being at low or high risk. The ELTS score was used to predicted long-term CML-related mortality in patients treated with a TKI.^9,10^ The Hammersmith score was used to evaluate the likelihood of the response to a second-generation TKI based on the prior response to imatinib, Sokal risk, and recurrence of grade 3 or 4 neutropenia; accordingly, patients were stratified into categories of good, intermediate, or poor risk.^11^

### Cytogenetic and molecular monitoring

Conventional cytogenetic analysis was performed on bone marrow samples, with ≥ 20 metaphases analyzed; the cytogenetic response was defined as the proportion of Philadelphia chromosome–positive metaphases among mitotic cells. Peripheral blood samples were collected longitudinally for molecular monitoring in accordance with ELN recommendations. The *BCR::ABL1* transcript levels were quantified using the GeneXpert^®^ system (Cepheid; Sunnyvale, California) via automated RT-qPCR (reverse transcription quantitative polymerase chain reaction). The results were reported using the International Scale, and internal quality controls were applied. This standardized approach enabled early and DMRs to be assessed throughout therapy.

### Response evaluation according to the ELN

#### Recommendations

Cytogenetic and molecular responses were evaluated according to the current ELN recommendations. A complete cytogenetic response (CCyR) was defined as the absence of Philadelphia chromosome–positive metaphases among mitotic cells. A major molecular response (MMR, MR3) was defined as *BCR::ABL1* ≤ 0.1% IS, MR4 as *BCR::ABL1* ≤ 0.01% IS, DMR as MR4 or deeper, and stable DMR as sustained deep molecular remission confirmed in ≥ 2 consecutive molecular assessments.^13,14,15^ Early molecular response was assessed at 6 and 12 months using ELN-defined milestones, and the best molecular response represented the deepest response achieved during therapy.

#### Survival endpoints

PFS was defined as the time from initiation of second-line TKI therapy to progression to the accelerated phase or blast crisis, treatment discontinuation due to disease progression, or death from any cause. OS was defined as the time from initiation of second-line TKI treatment to death from any cause.

#### Statistical analysis

Patient characteristics and treatment outcomes were summarized using descriptive statistics. Continuous variables are reported as medians with ranges, and categorical variables as frequencies and percentages. Univariate logistic regression was used to identify factors associated with achieving a DMR. Given the limited sample size, a parsimonious multivariate logistic regression model that included the 12-month ELN milestone, and ELTS risk and Hammersmith scores was constructed to avoid overfitting and collinearity; results are reported as odds ratios (ORs) with 95% confidence intervals (95% CIs). All statistical tests were two-sided, and p < 0.05 was considered statistically significant.

Kaplan–Meier analysis with the log-rank test was used to estimate survival. Confidence intervals were calculated using Greenwood’s formula with log–log transformation; in the small subgroups, the upper limits reached 100%. Landmark analyses at 6 and 12 months were used to estimate the 7-year OS by molecular response, excluding patients who died or were censored before each time point to avoid guarantee-time bias. Molecular milestones at 6 and 12 months were analyzed as fixed-time variables within these landmark analyses. In the additional analyses, the best molecular response achieved was considered to capture the overall depth of the response.

All statistical analyses were performed using MATLAB (version 2025b; The MathWorks Inc., Natick, Massachusetts) with the Statistics and Machine Learning Toolbox (version 25.2).

Approval for the study was granted by the Ethics Committee of the University Clinical Centre Niš, Niš, Serbia (approval number 19486/7, dated April 7th, 2025), and the research was performed according to the principles of the Declaration of Helsinki.

## RESULTS

### Patient characteristics and baseline risk stratification

The cohort consisted of 45 patients with chronic-phase CML who received nilotinib as second-line treatment. The median duration of prior imatinib therapy before switching to nilotinib was 27.3 months (range: 3–138). The main reasons for patients switching to the second-generation TKI were primary (44.5%) and secondary (40%) resistance, with a smaller proportion discontinuing imatinib treatment due to intolerance (15.5%). The baseline demographic and clinical characteristics of the study population are summarized in **Table 1**.

**Table 1:** Baseline characteristics and prognostic risk distribution of patients with chronic-phase chronic myeloid leukemia receiving nilotinib as second-line treatment

Characteristic	Variable	Number (%) or Median (range)
Age	Median, years	52.2 (20–85)
Sex, n (%)	Male/female	27 (60)/18 (40)
Imatinib therapy	Median, months (range)	27.3 (3–138)
Sokal score	Low Intermediate High Unknown	11 (24.5) 22 (48.9) 10 (22.2) 2 (4.4)
EUTOS score	Low High Unknown	38 (84.5) 5 (11.1) 2 (4.4)
ELTS score	Low Intermediate High Unknown	23 (51.1) 14 (31.1) 6 (13.3) 2 (4.4)
Hammersmith score	Good Intermediate Poor Unknown	24 (53.3) 12 (26.7) 7 (15.6) 2 (4.4)
Reason for second-generation TKI	Primary resistance Secondary resistance Intolerance	20 (44.5) 18 (40) 7 (15.5)

Percentages were calculated based on the total study population (N = 45). Unknown indicates missing baseline data. ELTS, EUTOS long-term survival; EUTOS; TKI, tyrosine kinase inhibitor.

Risk stratification according to the established prognostic scores showed that, based on the ELTS and Hammersmith scores, approximately half the cohort was classified as low risk (51.1%) and as good risk (53.3%), respectively. However, based on the Sokal score, intermediate-risk disease predominated (48.9%). Based on the EUTOS score, most evaluable patients were categorized as low risk (84.5%). The prognostic scores were calculated using the available baseline data.

### Cytogenetic and molecular response dynamics during second-line nilotinib therapy

The median duration of nilotinib therapy was 67.3 months (range: 6–150), enabling longitudinal assessment of cytogenetic and molecular responses. The response rates at the predefined time points are summarized in **Table 2**. The number of evaluable patients varied across the time points due to missing molecular assessments and treatment discontinuation.

**Table 2:** Cytogenetic and molecular responses during second-line treatment with nilotinib at predefined time points

Therapeutic response	6 Months, n/N (%)	12 Months, n/N (%)	Best response, n/N (%)
Cytogenetic response			
CCyR	37/45 (82.2)	35/41 (85.4)	40/45 (88.9)
Molecular response			
No MMR	14/32 (43.8)	10/38 (26.3)	10/45 (22.2)
MMR (without MR4)	10/32 (31.3)	13/38 (34.2)	8/45 (17.8)
DMR (MR4 or deeper)	8/32 (25)	15/38 (39.5)	27/45 (60)
Stable DMR	NA	NA	21/45 (46.7)

CCyR, complete cytogenetic response; MMR, major molecular response defined as *BCR::ABL1* ≤ 0.1% IS; DMR, deep molecular response defined as MR4 or deeper(*BCR::ABL1* ≤ 0.01% IS); stable DMR, stable deep molecular response defined as sustained MR4 or deeper confirmed on ≥ 2 consecutive assessments; NA, not applicable.

A CCyR was achieved in 82.2% of patients at 6 months and 85.4% of patients at 12 months, reaching a best response during therapy of 88.9%. An MMR or better (*BCR::ABL1* ≤ 0.1% IS) was observed in 56.3% of evaluable patients at 6 months’ this increased to 73.7% at 12 months, and 77.8% overall. The DMR (MR4 or deeper; *BCR::ABL1* ≤ 0.01% IS) showed a progressive accumulation over time, from 25% at 6 months to 39.5% at 12 months. This DMR reached 60%, forming the best molecular response. A stable DMR was documented in 46.7% of patients. Among patients who did not previously achieve a CCyR while undergoing imatinib treatment, 81.8% achieved a CCyR; among those who did not previously achieve an MMR, 59.3% achieved an MMR while undergoing second-line therapy.

### Performance of ELN: defined molecular milestones and prediction of DMR

Molecular responses were assessed according to the ELN recommendations. ELN-defined molecular milestones were achieved by 68.8% of evaluable patients at 6 months and 73.7% of patients at 12 months. Patients meeting these milestones showed higher rates of subsequent DMR than those who did not. Specifically, at last follow-up, a DMR was documented in 68.2% of patients who achieved the 6-month milestone versus 30% of those who did not. Similarly, a DMR was observed in 75% of patients who met the 12-month milestone versus 20% of those who did not.

### Univariate predictors of DMR

In the univariate logistic regression analysis, achieving the ELN-defined molecular milestones at 6 and 12 months was associated with an increased likelihood of achieving a DMR. Attaining the 12-month ELN milestone significantly increased the odds of achieving a DMR (OR, 12; 95% CI, 2.04–70.45; p = 0.006). A favorable Hammersmith score was also significantly associated with a DMR (OR, 11.08; 95% CI, 2.62–46.84; p = 0.001). Patients with low ELTS risk had a significantly higher probability of achieving a DMR compared with those at intermediate or high risk (OR, 0.24; 95% CI, 0.06–0.86; p = 0.028). Early cytogenetic and ­molecular responses at 6 months similarly correlated with subsequent DMRs. The wide confidence intervals observed for several estimates reflect the limited sample size and should be interpreted with caution.

### Multivariable analysis of predictors of DMR

A multivariate logistic regression model was constructed to identify independent predictors of DMR, including the 12-month ELN milestone, ELTS risk score, and Hammersmith score. Given the limited number of events, the model was restricted to these key variables to avoid overfitting. Achievement of the 12-month ELN milestone remained independently associated with a DMR (OR, 34.6; 95% CI, 2.56–467.67; p = 0.008), and a favorable Hammersmith score also independently predicted a DMR (OR, 14; 95% CI, 1.43–137.1; p = 0.023). By contrast, the ELTS risk category did not retain independent significance in the multivariate analysis, indicating that its predictive value was largely captured by the dynamic molecular response and Hammersmith score.

### Molecular responses, prognostic scores, and survival outcomes

The OS and PFS were analyzed as long-term clinical endpoints. At a median follow-up of 67.3 months, the 7-year OS and PFS for the entire cohort were 80.5% and 68.7%, respectively. Sex did not significantly influence survival, with a 7-year OS of 77.1% observed in males versus that of 94.4% observed in females (p = 0.148); the respective PFSs observed were 62.9% versus 77.5% (p = 0.116). Age at diagnosis was associated with OS: Patients younger than 65 years exhibited superior 7-year OS compared with those 65 years or older (89.8% versus 63%, respectively; p = 0.002), whereas PFS did not differ significantly (p = 0.107).

**Figure 1:**
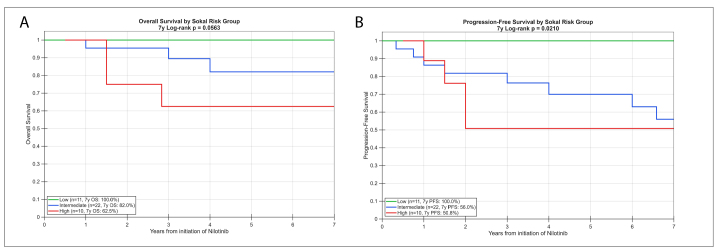
Overall and progression-free survivals according to the Sokal risk category. (a) The Kaplan–Meier curve for overall survival (OS) stratified by the Sokal risk category. (b) The Kaplan–Meier curve for progression-free survival (PFS) stratified by the Sokal risk category.

**Figure 2:**
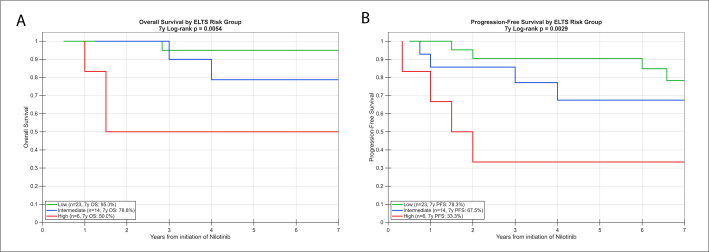
Overall and progression-free survivals according to ELTS risk category. (a) The Kaplan–Meier curve for overall survival (OS) stratified by ELTS risk category. (b) The Kaplan–Meier curve for progression-free survival (PFS) stratified by ELTS risk category.

Baseline prognostic scores demonstrated differential performance. The Sokal score was associated with PFS (7-year PFS: low, 100%; intermediate, 56%; and high, 50.8%; p = 0.021) but not OS (p = 0.056), whereas the ELTS score discriminated both OS (7-year OS: low, 95%; intermediate, 78.8%; and high, 50%; p = 0.005) and PFS (7-year PFS: low, 78.3%; intermediate, 67.5%; and high, 33.3%; p = 0.003) (**Figures 1 and 2**).

**Figure 3:**
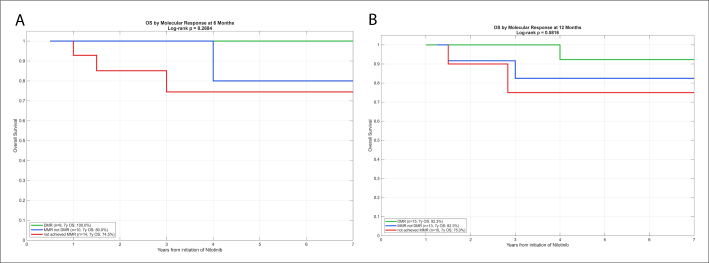
Landmark overall survival according to molecular response category. (a) The Kaplan–Meier curve for the 7-year overall survival (OS) stratified by molecular response (no major molecular response [MMR], MMR without deep molecular response [DMR], and DMR) at 6 months. (b) The Kaplan–Meier curve for the 7-year OS stratified by molecular response (no MMR, MMR without DMR, and DMR) at 12 months.

Landmark analyses at 6 and 12 months showed no statistically significant differences in OS according to molecular response at isolated time points. At 6 months, the 7-year OS was 74.5% in patients who did not achieve a MMR, 80% in those who achieved an MMR without DMR, and 100% in those who achieved a DMR (p = 0.268). At 12 months, the corresponding OS rates were 75%, 82.5%, and 92.3% (p = 0.582) (**Figure 3**).

**Figure 4:**
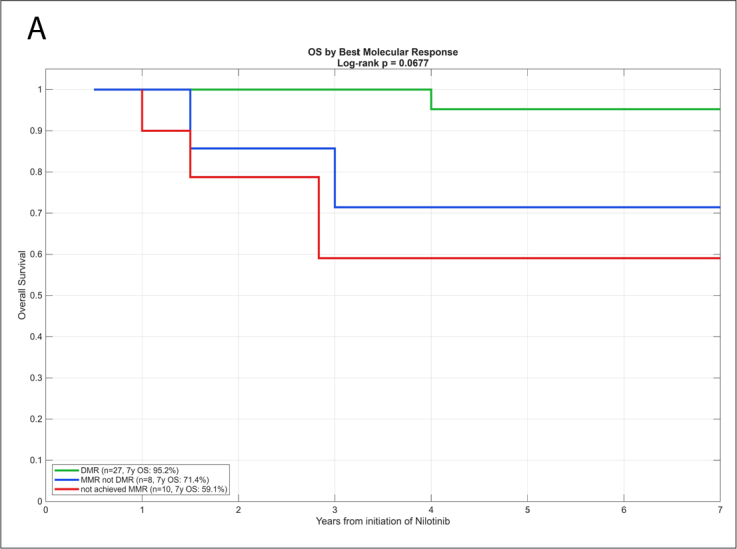
Overall survival (OS) according to best molecular response achieved during therapy. The Kaplan–Meier curve for the 7-year OS stratified by best molecular response category (no major molecular response [MMR], MMR without deep molecular response [DMR], and DMR).

The analysis based on the best molecular response achieved during therapy showed numerically different survival outcomes. Patients failing to achieve an MMR had a 7-year OS of 59.1%, those achieving an MMR but not DMR had an OS of 71.4%, and patients achieving a DMR had an OS of 95.2% (p = 0.068) (**Figure 4**). Although differences did not reach statistical significance, consistent numerical trends were observed across the response categories.

## DISCUSSION

Nilotinib, a second-generation TKI, selectively inhibits *BCR::ABL1* and demonstrates greater potency than imatinib, effectively overcoming most imatinib-resistant *BCR::ABL1* mutations.^16^ Clinical studies have shown that nilotinib is efficacious and generally well-tolerated in patients with imatinib-resistant or -intolerant disease, including those with resistance mechanisms independent of *BCR::ABL1* mutations.^16^ In newly diagnosed chronic-phase CML, nilotinib has been shown to reduce the emergence of resistant clones, suggesting a potential clinical advantage over imatinib.^17^ In this real-world cohort of patients with chronic-phase CML receiving second-line nilotinib treatment after treatment with imatinib failed, early ELN-defined molecular milestones and selected baseline prognostic scores were independently associated with subsequent DMRs. Real-world data integrating early molecular response with baseline prognostic scores to predict DMR remain limited, particularly for second-line use of nilotinib.

In the present study, longitudinal quantitative *BCR::ABL1* monitoring demonstrated that early attainment of ELN-defined molecular milestones at 6 and 12 months was strongly associated with subsequently achieving a DMR. Notably, the 12-month ELN milestone remained independently associated with DMR in the multivariable analysis, emphasizing the prognostic relevance of the dynamic assessment of response during treatment. Patients with prior imatinib resistance, including secondary resistance, achieved clinically meaningful rates of CCyR, MMR, and DMR, confirming the efficacy of second-line nilotinib use. In controlled clinical studies, MMR rates of approximately 73% and DMR rates of up to 34% were achieved within 2 years with the use of nilotinib as a second-line treatment, whereas real-world analyses reported cumulative MMR and DMR rates of approximately 65% and 40%, respectively, albeit with greater heterogeneity in the early response kinetics.^18,19,20,21,22^ The DMR rates observed in our cohort (60% best, 46.7% stable) align with these real-world data, reinforcing the effectiveness of second-line nilotinib treatment and supporting the use of early molecular milestones as predictive markers for deep remission.

In the modern era of multiple *BCR::ABL1* TKIs, DMRs and treatment-free remission have emerged as key therapeutic endpoints.^23^ However, most evidence on early *BCR::ABL1* transcript decline as a predictor of DMR is from frontline therapy, with limited data specifically addressing second-line use of nilotinib. Large real-world studies formally evaluating 6- or 12-month ELN milestones in this context remain lacking. Real-world data highlight the prognostic value of early molecular kinetics: Achieving MR4.5 was more likely with second-line use of nilotinib than dasatinib after adjustment for baseline characteristics.^24^ Furthermore, *BCR::ABL1* levels at 3 and 6 months independently predicted MR4, with higher rates of early molecular response being achieved with nilotinib use than with imatinib use.^25^ Early transcript dynamics within the first 3 to 6 months predicted subsequent DMR,^26^ whereas residual disease at 6 months was the strongest early predictor among the molecular parameters for deeper responses of MR4 or MR4.5.^27^ Real-world DMR rates with nilotinib generally remain lower than those reported in clinical trials, emphasizing the importance of contextualizing efficacy in routine practice.^28^ Collectively, these studies reinforce that early *BCR::ABL1* transcript kinetics during treatment provide robust predictive information for DMRs in second-line nilotinib therapy.

The results of the landmark analyses at isolated time points did not reach statistical significance for OS; however, the ­numerical gradients observed across the molecular response categories suggest that the cumulative depth of response may reflect long-term disease control. These observations should be interpreted cautiously due to the limited sample size. This aligns with prior studies highlighting that sustained DMR provides clinically meaningful information regarding durable disease control and potential treatment-free remission.^21,22,23^ Although ELTS risk stratification predicted DMR in the univariate analyses, its significance was attenuated in the multivariate models once molecular response during treatment and prior treatment sensitivity were included, confirming the dominant role of longitudinal molecular monitoring over baseline risk alone. Importantly, the ability of the ELTS score to discriminate overall and progression-free survivals was retained; however, the lack of independent significance for DMR observed for this score in the multivariable analysis suggests that dynamic molecular response during treatment provides more robust predictive information than baseline risk alone.

The integration of baseline prognostic scores with molecular response enabled additional stratification of patients who were at higher risk for suboptimal outcomes. Favorable Hammersmith scores were independently associated with DMR, consistent with baseline response-related factors, whereas dynamic kinetics during treatment were captured through ELN-defined molecular milestones; together, they provide complementary prognostic information.These observations are supported by prior studies in which baseline risk scores, particularly those for the ELTS, were shown to predict both survival and molecular outcomes in CML^29,30,31^; this highlights that integrating baseline and dynamic variables refines risk stratification and guides individualized monitoring.

From a clinical perspective, these results support the importance of achieving and maintaining DMRs in second-line nilotinib therapy. Our findings confirm the relevance of sustained DMR for long-term disease control and for the identification of patients who may be candidates for treatment-free remission. Integrating longitudinal molecular monitoring with baseline prognostic assessment and prior treatment sensitivity may help clinicians tailor monitoring intensity and identify patients most likely to achieve durable deep remission, thereby supporting individualized management in routine practice.

The limitations of this study include its retrospective, single-center design and modest sample size, which may limit statistical power and generalizability. Data on *BCR::ABL1* kinase domain mutations were not systematically available and thus were not included in the analysis. Future studies incorporating kinase domain mutation data are warranted to further refine predictors of DMR in second-line nilotinib therapy. The confidence intervals were wide for some predictors, reflecting the limited number of events. The survival analyses based on best molecular response may have been influenced by immortal time bias and should be interpreted cautiously. Nevertheless, by integrating baseline and dynamic variables, the findings of this study support a precision-oriented management approach to enhancing prognostic stratification and individualized monitoring even within a modest, single-center cohort.

## CONCLUSION

In this real-world cohort of patients with chronic-phase CML treated with second-line nilotinib, early *BCR::ABL1* kinetics combined with baseline prognostic scores provided complementary information for predicting DMR. Achievement of 12-month ELN milestones and a favorable Hammersmith score independently identified those patients most likely to reach a DMR, a key indicator of durable long-term disease control. Integrating longitudinal molecular monitoring with baseline risk enables clinicians to personalize follow-up and guide management in routine practice, reinforcing sustained DMR as a marker of durable disease control.

## Data Availability

Due to ethical and privacy restrictions, the raw data is not publicly available. The datasets generated and analyzed during the current study are available from the corresponding author, Irena Ćojbašić, on reasonable request.
